# The Rate of Negative Appendicectomy and Perforated Appendicitis As Quality Indicators of the General Surgical Service in a District General Hospital in Cavan, Republic of Ireland

**DOI:** 10.7759/cureus.39895

**Published:** 2023-06-03

**Authors:** Yousaf Tanveer, Yizhe Lim, Shumail Paulus, Muhammad Faheem Sarwar, Pawan Rajpal

**Affiliations:** 1 General Surgery, Cavan General Hospital, Cavan, IRL; 2 General Surgery, Craigavon Area Hospital, Craigavon, GBR

**Keywords:** morbidity, cost, united kingdom, ireland, general, outcomes, negative, appendicectomy, appendectomy, surgery

## Abstract

Introduction

Appendicitis is a common surgical condition that can be difficult to diagnose due to its varied clinical presentations. Surgical removal of the inflamed appendix is often necessary, and the appendix is sent for histopathological assessment to confirm the diagnosis. However, in some cases, the analysis may return a negative result for acute inflammation, known as a negative appendicectomy (NA). The definition of NA varies among experts. While negative appendicectomies are not ideal, they are accepted by surgeons to reduce the rate of perforated appendicitis, which can have severe consequences for patients. A study was conducted to investigate the rates of negative appendicectomies and their impact at a local district general hospital in Cavan, Republic of Ireland.

Methods

The study was conducted retrospectively from January 2014 to December 2019 on patients who were admitted with suspected appendicitis and underwent appendicectomy for appendicitis, regardless of age and sex. The researchers excluded patients who underwent elective, interval, and incidental appendicectomies. Data were collected on patient demographics, duration of symptoms prior to presentation, the intraoperative appearance of the appendix, and the histology results of the appendix specimens. Descriptive statistics and the Chi-squared test were used for data analysis with the help of IBM Statistical Package for the Social Sciences (SPSS) Version 26.

Results

The study retrospectively reviewed 876 patients who underwent an appendicectomy for suspected appendicitis between January 2014 and December 2019. The age distribution of patients was non-uniform, with 72% presenting before the third decade. The overall perforated appendicitis rate was 7.08%, and the overall negative appendicectomy (NA) rate was 21.3%. A subset analysis showed a statistically significant lower NA rate in females than in males. The NA rate decreased significantly over time and has been sustained since 2014 at around 10%, which is consistent with other published studies. The majority of the histology findings were uncomplicated appendicitis.

Discussion

This article discusses the challenges of diagnosing appendicitis and the need to reduce unnecessary surgeries. Laparoscopic appendectomy is the standard treatment, with an average cost of £2222.53 per patient in the UK. However, patients with negative appendicectomies (NA) have longer hospital stays and higher morbidity than uncomplicated cases, making it crucial to reduce unnecessary surgeries. The clinical diagnosis of appendicitis is not always straightforward, and the rate of perforated appendicitis increases with a longer duration of symptoms, particularly pain. The selective use of imaging in suspected appendicitis could reduce NA rates, but a statistical difference has not been proven. Scoring systems like the Alvarado score have limitations and cannot be relied upon alone. Retrospective studies have limitations, and biases and confounding variables must be considered.

Conclusion

The study found that a thorough investigation of patients, particularly with preoperative imaging, can decrease the rate of unnecessary appendectomies without increasing the rate of perforation. This could save costs and reduce harm to patients.

## Introduction

Appendicitis is a common condition encountered in general surgery that requires prompt treatment to prevent complications. In many cases, surgical removal of the inflamed appendix, known as an appendicectomy, is necessary. However, there are challenges in diagnosing appendicitis due to its varied clinical presentations, and differentials may include non-specific abdominal pain, tubo-ovarian pathology, and mesenteric adenitis [1,2]. This can make it difficult to confidently proceed with surgery without detailed investigations to confirm the diagnosis.

Following surgery, the appendix is typically sent for histopathological analysis to confirm the diagnosis and rule out other potential causes of inflammation, such as malignancy. However, in some cases, the analysis may return a negative result for acute inflammation, known as a negative appendicectomy (NA). There is no consensus on what is NA, with some considering a histologically normal appendix only, with no pathology [3]. Some authors have described a more specific definition of "the absence of intramural neutrophils in the appendix [4]." The latter is what we have used in this study.

While negative appendicectomies are a compromise, general surgeons must accept them to reduce the rate of perforated appendicitis, which can have a significant impact on patient outcomes. In our study, we aimed to investigate the rates of negative appendicectomies and their impact at the Cavan General Hospital, our local district general hospital in Cavan, Republic of Ireland.

## Materials and methods

We retrospectively reviewed patients admitted with suspected appendicitis from January 2014 to December 2019. Our inclusion criteria are patients that have undergone an appendicectomy for appendicitis, regardless of age and sex.

Through electronic health records, we have identified patients who underwent either a laparoscopic or open appendicectomy. Reviewing their operation notes, we excluded patients who underwent elective, interval, and incidental appendicectomies.

We collected data regarding demographics (i.e., sex, age) through their health records and reviewed their history specifically for the duration of symptoms prior to presentation. We then looked through their operation notes for the intraoperative appearance of the appendix and classified them into uncomplicated appendicitis, perforated appendicitis, and normal appearance. We then looked through the appendix specimens sent for histology, breaking down the results into negative (based on previous criteria), appendicitis (early and acute), and neoplasm.

The data were then tabulated in Microsoft Excel and analysed using descriptive statistics, and the Chi-squared test was employed for any statistical significance with the help of IBM Statistical Package for the Social Sciences (SPSS) Version 26.

## Results

**Figure 1:**
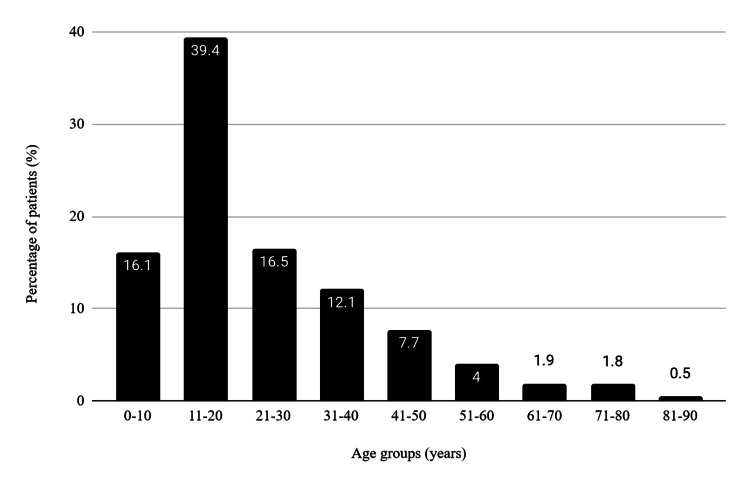
Age distribution of patients presenting with suspected appendicitis

We identified a total of 876 patients who underwent an appendicectomy for suspected appendicitis. There were around the same number of males and females (n=424, n=452). The age distribution of patients was non-uniform, with 72% of patients presenting before the third decade. This is further illustrated in Figure 1.

During laparoscopy, the findings were divided into perforated appendicitis (PA) and uncomplicated appendicitis, as well as those that appeared normal. The overall PA rate was 7.08%.

We excluded the ones that appeared normal and compared the difference in perforations between males and females, which is broken down in Table 1. This was not found to be statistically significant (p=0.231).

**Table 1:** The number of perforated appendicitis and uncomplicated appendicitis mapped according to the sex of the patients

	Perforated appendicitis	Uncomplicated appendicitis
Males	36	206
Females	26	226

With histopathology, there were some incomplete reports, which left us with n=777 reports that we could analyse. Using histology as the final diagnosis, the majority of our patients had appendicitis (n=599, 77.2%), with a small number having appendiceal neoplasms. This is broken down in Table 2.

**Table 2:** The breakdown of the histopathology findings of appendixes sent for histopathological testing

Histology finding	Number (%)
Negative	166 (21.3%)
Uncomplicated appendicitis	432 (55.7%)
Complicated appendicitis (gangrenous and perforated)	167 (21.5%)
Neoplasm	12 (1.5%)

Our overall NA rate was 21.3%. A subset analysis to compare males and females was performed and was found to be statistically significant (p<0.001). The data used are summarised in Table 3.

**Table 3:** Breakdown of negative appendixes and appendicitis on histology (i.e., uncomplicated and complicated) according to the sex of the patients

Sex	Negative	Appendicitis
Male	56	206
Female	110	227

**Figure 2:**
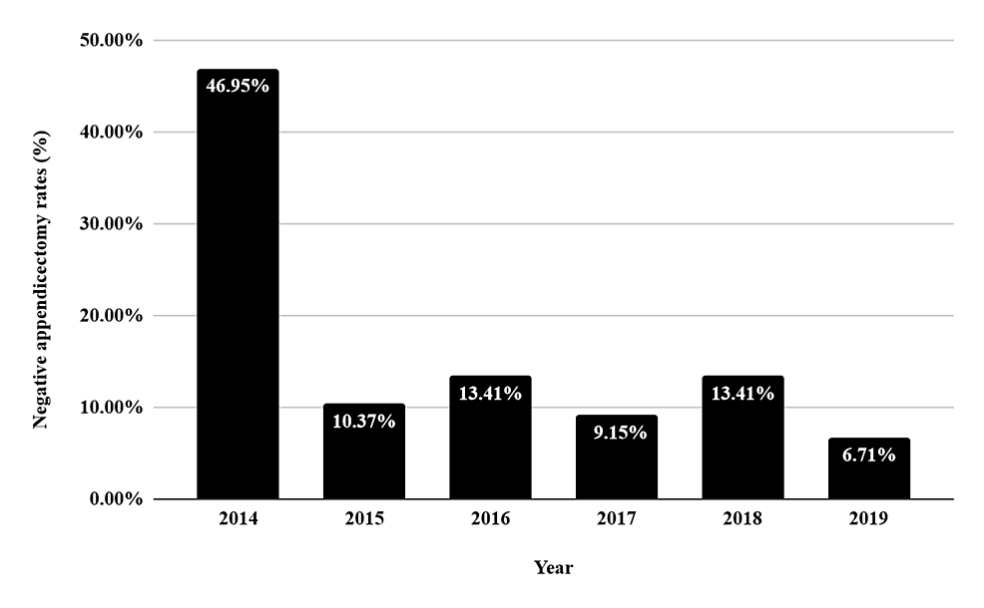
Negative appendicectomy rates over time at our centre

Measuring the rates of NA over time, we noticed a significant and sustained decline since 2014, when the audit was done in our unit. Just under half of all appendicectomies were negative, or around 10%. This is highlighted in Figure 2.

## Discussion

Appendicitis is a common cause of right iliac fossa pain and is associated with a significant burden and cost to healthcare systems, such as the National Health Service (NHS) in the UK. Laparoscopic appendicectomy is the standard treatment for patients with suspected appendicitis, with an average cost of £2222.53 per patient [5]. The NHS performs 50,000 appendicectomies annually [6], which accounts for around £111 million annually. It has also been reported that patients with NA have longer hospital stays than patients with uncomplicated appendicitis, which increases the total cost [7] and may have the potential to reduce the cost of the health service.

The clinical diagnosis of appendicitis is not always straightforward, and there is pressure on the general surgeon to decide on an operation to avoid perforated appendicitis (PA). The rate of PA is estimated to be between 16% and 30% at presentation and increases by 5% for every 12 hours that elapse after 36 hours from the onset of symptoms [1]. Our study found a lower rate of PA than published estimates but also found that the rate also increased with a longer duration of symptoms, particularly pain.

Traditionally, high NA rates were thought to reduce the incidence of PA, with NA rates between 25% and 35% being considered acceptable [3]. However, in recent years, there has been a shift to lower NA rates, which have been used as a quality indicator for surgical services [2,3], reflecting the thorough investigations of patients, particularly in cases of diagnostic uncertainty [3].

At our centre, our overall NA rate is consistent with the rest of the UK, with a rate of 21.3% compared to the national average of 20.6% [8]. However, this is higher than in other developed nations, such as the United States of America (USA), where the NA rate is between 5% and 10% [9]. The difference is attributed to the more routine and judicious use of preoperative imaging, particularly computed tomography (CT), in the USA [9], whereas the UK still uses a mixed and conservative approach to imaging. Since the audit was first carried out in 2014, we have seen a decrease in our NA rate to a more acceptable range of 6.7%-13.4%. We attribute this to the more frequent use of preoperative imaging, which allows a reduced exposure to unnecessary surgery without a significant increase in the rate of PA.

Performing a laparoscopic appendicectomy also carries higher morbidity for patients with NA than uncomplicated appendicitis [10]. This is measured in terms of infections (both superficial and deep), cardiorespiratory complications, and intestinal complications (ileus and obstruction). Coupled with a longer length of stay (LOS) [7,10], patients are subjected to surgical risks and postoperative morbidity without any objective improvement and have a potential for harm. This is of greater importance seeing that the majority of patients with NA are children, particularly young girls with tubo-ovarian pathology [11]. Moreover, removing the vermicular appendix in children, with or without appendicitis, increases the risk of adult cardiovascular and cerebrovascular events [12].

The selective use of imaging in suspected appendicitis could, in theory, reduce NA rates without exposing a greater number of the population to radiation. However, a statistical difference in NA rates between routine and selective imaging has not been proven [13]. Females are statistically more likely to have NA, both in our study and published literature, particularly in women of reproductive age (WORA) [10,14-16]. Even strong suspicion of appendicitis would transpire in confirmed appendicitis in around a third of the patients [14], highlighting the need for careful investigation in this group. Supporting this, routine CT significantly reduces the need for appendicectomy in females under 45 years old, more so than those above 45 years old, when appendicitis is more likely than gynaecological pathology [9,15,16]. However, routine CT has not been shown to reduce NA rates for males of any age [9,15,16].

Scoring systems, in particular the Alvarado score, have been used to strengthen the diagnosis of appendicitis without imaging [17]. An Alvarado score of seven or greater correlates more with positive pathology in patients over 16 years of age [18], but it should not be used as a diagnostic tool on its own as it is not reliable enough, especially when compared to cross-sectional imaging such as CT [15,16]. Moreover, the Alvarado score is inconsistent among children [15,16] and over-predicts the probability of appendicitis in women [15,16], which limits its usefulness in daily clinical practice.

Finally, it is important to acknowledge the limitations of our study. By design, it is a retrospective study, and despite our best efforts to gather as much information as possible, there were cases where we were unable to obtain complete records for some patients. This may have influenced our findings and limited the overall scope of our study. In addition to other factors like bias and confounding variables, our study would be level III evidence.

## Conclusions

Our study has demonstrated a reduction in rates of negative appendicectomies without a rise in perforated appendicitis in our centre through careful preoperative assessment, in particular the use of preoperative imaging, particularly computed tomography. This has cost-saving implications as well as reducing the risk of harm to patients.
